# Bleaching Gels Containing Calcium and Fluoride: Effect on Enamel Erosion Susceptibility

**DOI:** 10.1155/2012/347848

**Published:** 2012-10-30

**Authors:** Alessandra B. Borges, Carlos R. G. Torres, Paulo A. B. de Souza, Taciana M. F. Caneppele, Luciana F. T. F. Santos, Ana Carolina Magalhães

**Affiliations:** ^1^Department of Restorative Dentistry, School of Dentistry, Universidade Estadual Paulista (UNESP), São José dos Campos, Av. Francisco José Longo, 777, 12245-000 São José dos Campos, SP, Brazil; ^2^Department of Biological Sciences, Bauru School of Dentistry, University of São Paulo (USP), Alameda Octavio Pinheiro Brisola, 9-75, 17012-901 Bauru, SP, Brazil

## Abstract

This *in vitro* study evaluated the effect of 35% hydrogen peroxide (HP) bleaching gel modified or not by the addition of calcium and fluoride on enamel susceptibility to erosion. Bovine enamel samples (3 mm in diameter) were divided into four groups (*n* = 15) according to the bleaching agent: control—without bleaching (C); 35% hydrogen peroxide (HP); 35% HP with the addition of 2% calcium gluconate (HP + Ca); 35% HP with the addition of 0.6% sodium fluoride (HP + F). The bleaching gels were applied on the enamel surface for 40 min, and the specimens were subjected to erosive challenge with Sprite Zero and remineralization with artificial saliva for 5 days. Enamel wear was assessed using profilometry. The data were analyzed by ANOVA/ Tukey's test (*P* < 0.05). There were significant differences among the groups (*P* = 0.009). The most enamel wear was seen for C (3.37 ± 0.80 **μ**m), followed by HP (2.89 ± 0.98 **μ**m) and HP + F (2.72 ± 0.64 **μ**m). HP + Ca (2.31 ± 0.92 **μ**m) was the only group able to significantly reduce enamel erosion compared to C. The application of HP bleaching agent did not increase the enamel susceptibility to erosion. However, the addition of calcium gluconate to the HP gel resulted in reduced susceptibility of the enamel to erosion.

## 1. Introduction

Tooth whitening is a highly desirable esthetic treatment, as the tooth color is one of the most important factors related to the patients' satisfaction with their appearance [1].

Dental bleaching treatments are mainly based on the action of hydrogen peroxide, which is able to penetrate the tooth structure and release free radicals, oxidizing the chromophore molecules. Such molecules are mainly organic, although inorganic molecules can also be affected by these reactions [2]. Nevertheless, the free radical reaction is not specific and it may also alter the organic component of enamel [3]. Since the organic content contributes to the integrity of enamel, different adverse effects on both mineral and organic parts of bleached enamel have been observed [3].

Alterations in enamel surface morphology [3–6], chemical composition [7–10], and microhardness values [3, 11, 12] after bleaching were previously reported. Furthermore, some changes in bleached enamel were also described as slight erosive effects promoted by the bleaching agent [6, 13]. Nevertheless, some authors claim that the erosive pattern on the surface of bleached enamel only occurs when bleaching gels with low pH are used [14, 15]. 

Attempts to minimize the adverse effects of bleaching treatments by increasing enamel remineralization have been conducted, however, the results are contradictory. De Oliveira et al. [16] observed no significant increase of bleached enamel microhardness when calcium and fluoride were added to 10% carbamide peroxide gel. On the other hand, Borges et al. [12] observed a significant increase of enamel microhardness after bleaching with 35% hydrogen peroxide agent with the addition of calcium and fluoride. Chen et al. [6] also reported a less distinct erosion pattern on the surface of enamel bleached with fluoridated gels.

The association between the bleached enamel surface alterations and the subsequent susceptibility to erosive lesions resulting from the contact of bleached enamel with demineralizing solutions has been discussed. In a previous study, the application of carbamide peroxide gel rendered enamel more susceptible to demineralization [17]. In other studies, at-home bleaching technique did not increase the susceptibility of enamel to erosion [18, 19]. However, the effect of at-office bleaching agent (35% HP) on the enamel susceptibility to erosion has not been properly discussed. 

Considering the possibility that bleaching gels with high concentration of HP could increase the susceptibility of enamel to erosion, the addition of remineralizing ions into bleaching gels would be beneficial for preventing a further enamel demineralization [6].

Therefore, the objective of this *in vitro* study was to evaluate the effect of bleaching agents based on 35% hydrogen peroxide modified or not by the addition of calcium or fluoride on the susceptibility of enamel to erosion. The null hypotheses tested were (1) 35% HP bleaching agent does not increase the susceptibility of enamel to erosive challenges and (2) there is no difference in erosion susceptibility of enamel that has been treated with hydrogen peroxide compared to enamel treated with hydrogen peroxide supplemented with calcium or fluoride.

## 2. Materials and Methods

### 2.1. Preparation of the Samples

Freshly extracted nondamaged and intact bovine incisors were stored in a 0.1% thymol solution and refrigerated at 4°C, until required. Cylindrical enamel samples (3 mm in diameter and 1 mm height) were prepared from the labial surface of the tooth using a trephine mill (Dentoflex, São Paulo, SP, Brazil). The enamel surface was ground flat and polished with water-cooled silicon carbide (SiC) paper discs (1200, 2500, and 4000 grit; Fepa-P, Panambra, São Paulo, SP, Brazil), thereby removing approximately 200 *μ*m of the outermost layer as verified with a micrometer (Micromar 40EXL, Mahr-Goettingen, Germany).

The specimens were immersed in deionized water and placed in an ultrasonic bath for 10 min (Ultrasonic Cleaner, Odontobras, Ribeirao Preto, Brazil) for the removal of all waste, and then stored in thymol solution at 0.1% for rehydration.

**Figure 1:**
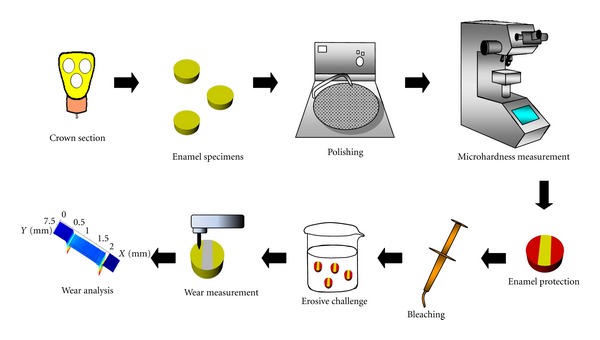
Schematic representation of sample preparation and analysis.

After polishing, the enamel specimens were selected from the average of the surface microhardness measured using a Microhardness tester (FM-700, Future-Tech, Tokyo, Japan-Knoop tip, average of three indentations, under 25 g-load for 10 s) with an allowable variation within 20% of the mean. Microhardness average of each specimen was used for stratified allocation among 4 groups (*n* = 15), so that the average microhardness for each group was similar. In order to maintain the reference surfaces for lesion-depth determination (profilometry), 2 layers of nail varnish were applied on 2/3 of the surface of each sample (1/3 of each side) exposing a central band area (see Figure 1).

### 2.2. Bleaching Procedures

The groups were divided as follows: C (control)-nonbleached, HP-bleached with 35% hydrogen peroxide gel without addition of remineralizing agents (pH = 6.35), HP + Ca-bleached with 35% hydrogen peroxide gel with the addition of 2% calcium gluconate (pH = 7.99), and HP + F-bleached with 35% hydrogen peroxide gel modified by the addition of 0.6% sodium fluoride (pH = 8.11).

The experimental groups were subjected to 35% hydrogen peroxide-based bleaching agents and modified by the manufacturer (based on Whiteness HP Blue formulation-FGM Dental Products, Joinville, SC, Brazil). The pH of the agents was measured using a pH meter (Digimed DM-20-Digicrom Analítica Ltda., São Paulo, Brazil) fitted with an electrode (DME-Digimed CV8) that was calibrated using solutions with pH 4.01 and 6.86.

A layer of approximately 2 mm of the bleaching gel was applied on the enamel surface for 40 minutes. The gel was periodically shaken to remove the air bubbles formed during the procedure. After this period, the specimens were rinsed with deionized water to remove the bleaching agent (20 s).

### 2.3. Erosive Challenge

After the bleaching procedures, the enamel samples were immersed in artificial saliva for 2 h and then subjected to erosive challenges for 5 days. The erosive cycles consisted of immersion in unstirred soft drink (20 mL/sample, Sprite Zero, Companhia Fluminense de Refrigerantes, Porto Real, RJ, Brazil), pH 2.8, four times per day for 2 min each time [20], followed by a remineralizing period of 2 h between erosive challenges (immersion in unstirred artificial saliva, pH 7.0, 20 mL/sample), at room temperature in small containers. The samples were kept in artificial saliva overnight. The composition of the artificial saliva was the same as used by Göhring et al. [21]: hydrogen carbonate (22.1 mmol/L), potassium (16.1 mmol/L), sodium (14.5 mmol/L), hydrogen phosphate (2.6 mmol/L), boric acid (0.8 mmol/L), calcium (0.7 mmol/L), thiocyanate (0.4 mmol/L), and magnesium (0.2 mmol/L). The pH was adjusted to 7.0 using concentrated HCl. 

The beverage was replaced at the end of each challenge, and artificial saliva was renewed daily.

### 2.4. Surface Wear Assessment

Profiles were obtained from the enamel surfaces with a profilometer (MaxSurf XT 20, Mahr-Goettingen, Germany) after the erosive challenge. To determine the change in the surface profile after the experiment, the nail varnish was carefully removed with a spatula and a solution of acetone (1 : 1-acetone : water) to clean the surface. The diamond stylus moved from the first reference to the exposed area and then over to the other reference area (2.5 mm long and 1.0 mm wide). The vertical distance between the horizontal line drawn on the reference areas and the horizontal line drawn on experimental area was defined as tooth wear using the software (Software Mahr Surf XT20, 2009). Five profile measurements were performed for each specimen at intervals of 0.25 mm and the values were averaged (*μ*m).

A schematic representation of the sample preparation and analysis is presented in Figure 1. 

### 2.5. Statistical Analysis

Assumptions of normal distribution of error and equality of variance (Kolmogorov-Smirnov and Bartlett's tests) were checked for all the variables tested. Since the assumptions were justified, one-way ANOVA followed by post hoc Tukey's test were used to compare the different bleaching agents in relation to the susceptibility of enamel to erosive wear. Statistical analysis was performed with the software Statistica for Windows (Statsoft, Tulsa, OK, USA), with a significance level of 5%.

## 3. Results

 One-way ANOVA revealed significant differences between the groups (*P* = 0.009).  Table 1 shows the results of post hoc Tukey's test. The bleaching groups HP, HP + F, and HP + Ca, presented similar enamel wear values among them (n.s.). However, specimens from group HP + Ca had significantly less mean enamel wear compared to the control after the erosive challenges, while the groups HP and HP + F did not differ from the control. 

## 4. Discussion

Although in-office bleaching is considered a short-term treatment, it should not represent an additional risk for subjects susceptible to erosion. In the present study, the exposure of 35% HP-bleached enamel to the acid beverage did not cause higher enamel surface wear compared to control group (unbleached). Therefore, the first null hypothesis tested was accepted. It may be suggested that possible microstructural changes caused by the bleaching treatment did not predispose the enamel to greater erosive wear. These microstructural changes may have been repaired by the adsorption and precipitation of salivary calcium and phosphate, when immersed in artificial saliva for 2 h before the erosive challenge [17]. The ability of saliva to reharden and replenish lost minerals from bleached enamel has been demonstrated previously [22, 23]. 

Although the adverse effects of bleaching agents on enamel are mainly attributed to the concentration and pH of the bleaching gel [14, 24], some surface alterations are reported even with low-concentrated and nonacidic agents [4, 5, 9]. In fact, the demineralization of bleached enamel can occur as a result of mineral and organic content alterations [4, 5, 25]. Even though these factors are considered reversible with exposure to saliva [22], it might be speculated that when the bleached enamel is exposed to acid, these minimal changes could promote a greater spread of erosive agents inside enamel, leading it to a more pronounced demineralization [17]. 

Previous studies have shown that bleached enamel exposed to 37% phosphoric acid, which is applied before bonding procedures, presented a higher acid dissolution, with increased amount of Ca^2+^ extracted from enamel and an uneven etched surface, compared to the unbleached enamel [26, 27]. Nevertheless, other authors only reported this increased decalcified effect when high-concentrated hydrogen peroxide was used [28]. 

The association between carbamide peroxide bleaching and erosion and abrasion were previously investigated. Pretty et al. [18] applied 20 cycles of bleaching (2 h) using carbamide peroxide gels, from 10 to 22% concentration, and brushing (2 min), on enamel surface. The specimens were then immersed in 0.1% citric acid for a total of 14 h. The authors found no increased risk of enamel to acid dissolution. Engle et al. [19] also failed to demonstrate a significant increase in the susceptibility of bleached enamel to erosion/abrasion after a 5 days-bleaching treatment with 10% carbamide peroxide (10 h/day) associated with erosive and abrasive challenges. Although the bleaching agent tested in this study was more concentrated, which could increase the adverse effects, the unique application time was shorter (40 min) and, thus, similar results were achieved compared to control. 

This study simulated only one session of bleaching treatment, however, multiple appointments are needed for obtaining optimal bleaching outcomes [29]. Therefore, the subsequent erosive wear of bleached enamel could be more evident after successive bleaching, as more severe chemical alterations have been observed in enamel bleached with high-concentrated hydrogen peroxide for long periods of exposure [30]. On the other hand, it might be speculated that the alterations provoked by the bleaching agents could be relevant only for the susceptibility of the enamel to initial erosive lesions (“erosion” phase), in which enamel softening, but not wear, is seen, as observed previously [17]. 

This experiment was conducted to simulate the effect of the intake of soft drinks (Sprite Zero that has no potential to stain the bleached enamel) after a session of in-office bleaching treatment using a high-concentrated hydrogen peroxide gel. The use of bovine enamel is considered a convenient substitute for human enamel in erosion studies, as they are easier to obtain, to handle, and standardize. Nevertheless, it has to be considered that these two substrates can behave in different physical and chemical manners and it was shown that the demineralization occurs faster in bovine enamel, due to its greater porosity [31–33].

 The addition of potentially remineralizing agents in bleaching gels was also investigated in this study. The calcium and fluoride were added to the thickener phase of the gel, resulting in a concentration of 2% and 0.6%, respectively, after the final mixture, as these concentrations were compatible with the chemical formulation of the gel, without impairing its thickness. It would be conceivable that the bleaching gels containing remineralizing agents could act not only as whitening agents but also as remineralizing agents [34]. Supplements of fluoride and calcium in the bleaching gels have been shown to minimize the deleterious effects on enamel. It is supposed that the saturation of these ions in the gel allow their incorporation into the enamel apatite, increasing the resistance to demineralization [12, 35]. 

It was previously reported that the application of fluoride gel after bleaching resulted in increased resistance of the enamel against erosive attacks, compared to bleached/unfluoridated enamel [36]. However, when fluoride was added to the bleaching gel, the protection against demineralization was reported to be limited compared to fluoride only, since peroxide reduced the fluoride uptake by bleached enamel [34]. 

In the present study, the erosive challenges tested were frequent (4 cycles of 2 min each per day, for 5 days); therefore, the presence of fluoride into gel might have not been able to provide protection against enamel wear. As a reduced formation of KOH-soluble fluoride was observed in enamel bleached with fluoridated agents [34], it can be speculated that any “calcium fluoride-like” precipitation was readily dissolved by the subsequent acid challenges [37].

In addition, it has to be considered that fluoride dentifrices are frequently used in the daily practice and this additional source of fluoride can eventually contribute remineralizing the enamel surface after the bleaching procedures and erosive challenges. Nevertheless, the fluoride dentifrice was not included in this experimental model as this study attempted to analyze the effect of remineralizing agents added to the bleaching gel. Furthermore, fluoride dentifrice application is usually associated with a brushing procedure, which may increase the enamel erosive wear [38]. Due to the fact that the focus of this study was only on enamel susceptibility to erosion, brushing with fluoride dentifrice was not included in the pH cycling model. 

In contrast to the effect of adding fluoride into the bleaching gel, the addition of calcium gluconate to the bleaching gel resulted in a protective effect against the erosion; thus, the second null hypothesis was rejected. In a previous study, deposits of calcium on the surface of the enamel bleached with calcium-added agent were shown using scanning electron microscope analysis [23]. These deposits may have acted as a physical barrier, minimizing the contact of the acid to enamel, or providing additional mineral to be dissolved during the acid challenge before the underlying enamel was attacked. Another forms of calcium combined with bleaching agents have been previously investigated, such as the casein phosphopeptide-amorphous calcium phosphate (CPP-ACP) [39, 40] and calcium chloride [12], resulting in increased mechanical properties of bleached enamel.

Nevertheless, future studies should be conducted to investigate the solubility of different presentation forms of calcium salts, as well as their interaction with enamel surface, so that the mechanism of action of calcium in improving the resistance of bleached enamel to erosion can be established in different phases of its development (erosion and erosive wear). 

## 5. Conclusion

Considering the experimental design, it can be concluded that the application of 35% HP-based bleaching agents did not alter the enamel susceptibility to erosion, and the addition of calcium to the bleaching gel improved erosion resistance of the bleached enamel.

## Figures and Tables

**Table 1:** Mean (standard deviation) of the erosive enamel wear (*μ*m) for the different tested groups.

	Mean (standard deviation)	Homogeneous Sets*
C	3.37 (0.80)	a
HP	2.82 (0.98)	ab
HP + F	2.72 (0.64)	ab
HP + Ca	2.31 (0.92)	b

^∗^Groups with different letters showed significant differences between them (*P* < 0.05).
